# Inverting sediment bedforms for evaluating the hazard of dilute pyroclastic density currents in the field

**DOI:** 10.1038/s41598-021-00395-3

**Published:** 2021-10-25

**Authors:** Pierfrancesco Dellino, Fabio Dioguardi, Anna Rinaldi, Roberto Sulpizio, Daniela Mele

**Affiliations:** 1 Dipartimento Di Scienze Della Terra E Geoambientali, Università Di Bari, Bari, Italy; 2 British Geological Survey, The Lyell Centre, Edinburgh, UK; 3 Dipartimento Di Economia E Finanza, Università Di Bari, Bari, Italy

**Keywords:** Natural hazards, Solid Earth sciences

## Abstract

Pyroclastic density currents are ground hugging gas-particle flows associated to explosive volcanic eruptions and moving down a volcano's slope, causing devastation and deaths. Because of the hostile nature they cannot be analyzed directly and most of their fluid dynamic behavior is reconstructed by the deposits left in the geological record, which frequently show peculiar structures such as ripples and dune bedforms. Here, a set of equations is simplified to link flow behavior to particle motion and deposition. This allows to construct a phase diagram by which impact parameters of dilute pyroclastic density currents, representing important factors of hazard, can be calculated by inverting bedforms wavelength and grain size, without the need of more complex models that require extensive work in the laboratory.

## Introduction

Geologists have always been fascinated by sediment bedforms. They are a natural beauty of practical importance and represent helpful insights in the reconstruction of ancient sedimentary environments^1^. They form in a range of directional currents such as aeolian, fluvial, turbiditic flows, snow avalanches and pyroclastic density currents (PDCs). When a current flowing over sediment exceeds the critical shear stress for motion, bedforms develop as a result of the interaction between sediment and fluid^2^. The bedforms developing at the lowest speed are called ripples^2^ and have wavelengths, (*λ*), smaller than 60 cm. Larger bedforms are called dunes^3^. It is widely recognized that the occurrence of ripples or dunes depends on hydrodynamic conditions and sediment characteristics. These are defined in phase diagrams^4,5^ where bedform characteristics as wavelength and particle-size characteristics as the median size, (*D)*, are related to flow parameters, as the densiometric Froude number $${(Fr}^{^{\prime}})$$^6^ and the critical Shields number $${(\theta }_{t})$$^7^_,_ the former being a balance between inertial and gravitational effects, the latter representing the threshold of initiation of motion, which is a function of the Reynolds’ number of shear ($${Re}_{*})$$^1^ and for pyroclastic materials was measured through wind tunnel experiments using pumice and scoria as a function of grain size and bed slope^8^. For symbols definitions see Table1.

**Table 1:** List of symbols, with description and physical dimension.

Symbol	Description	Dimension
*A*_*r*_	Aggradation rate per unit width	ms^−1^
*C*	Particle volumetric concentration	–
*C*_*0*_	Reference known concentration (0.75)	–
*C*_*sf*_	Depth-averaged concentration in the basal shear flow	–
*C*_*d*_	Particle drag coefficient	–
*D*	Sediment median size	mm
*Fr’*	Froude number—${Fr}^{^{\prime}}=\frac{V}{\sqrt{{g}^{^{\prime}}H}}$	
*g*	Gravity acceleration (9.81)	ms^−2^
*g’*	Reduced gravity—${g}^{^{\prime}}=g\left(\frac{{\rho }_{mix}-{\rho }_{f}}{{\rho }_{f}}\right)$	ms^−2^
*H*	Current depth	cm
*H*_*dep*_	Deposit thickness	cm
*k*	Von Karman constant (0.4)	–
*k*_*s*_	Substrate roughness	cm
*P*_*dyn*_	Dynamic pressure	Pa
*P*_*n*_	Particle Rouse number	–
*P*_n_*	Normalized Rouse number	–
*P*_*navg*_	Average Rouse number of solid material	–
*P*_*ni*_	Rouse number of the ith particle-size class	–
*P*_*nsusp*_	Rouse number at maximum suspension capacity	–
*Q*_*b*_	Bedload transportation rate	m^2^s^−1^
*q*_*bi*_	Volumetric bedload transport rate of the ith particle-size class	m^2^s^−1^
Re_*_	Reynolds’ number—${Re}_{*}=\frac{{\rho }_{mix}{u}_{*}D}{\mu }$	–
*S*_*r*_	Sedimentation rate	kgm^−2^ s^−1^
*S*_*rw*_	Sedimentation rate per unit width	m^2^s^−1^
*t*	Flow duration	s
u_*_	Shear velocity	ms^−1^
*V*	Current velocity	ms^−1^
*W*_i_*	Dimensionless transport rate of the ith particle-size class	–
*w*_*t*_	Particle terminal velocity	ms^−1^
*w*_*ti*_	Terminal velocity of the ith particle-size class	ms^−1^
*y*	Flow vertical coordinate	cm
*y*_*0*_	Specific height of C_0_	–
α	Slope angle	
ϕ	Unit of grain-size distribution ( ϕ = −*log*_*2*_*d; d is in mm*)	–
ϕ_*i*_	Weight fraction of the i_th_ size class	Weight%
*θ*_*t*_	Shield’s number—${\theta }_{t}=\frac{{\rho }_{mix}{u}_{*}^{2}}{Dg({\rho }_{s}-{\rho }_{mix})}$	–
λ	Wavelength	cm
μ	Fluid viscosity	Pas
ρ_*f*_	Fluid density	kgm^−3^
ρ_*mix*_	Density of the fluid-particle mixture	kgm^−3^
ρ_*s*_	Particle density	kgm^−3^
ρ_*sf*_	Density of shear flow	kgm^−3^
ρ_*si*_	Density of the ith particle-size class	kgm^−3^
τ	Shear stress at the base of the current	Pa
τ_*ri*_	Minimum shear of the *i*_*th*_ size fraction	Pa
ξ	Normalized shear stress	–

PDCs form upon explosive eruptions when mixtures of gas, and fragments of magma and lithics, ranging in size from ash to blocks and bombs, collapse from vertical eruption columns or are generated from gravitational failure of domes^9^. They feed flows that may spread around the volcano for many kilometers severely impacting infrastructures and people^10^. Our understanding of PDCs relies primarily on the information preserved in the sediments of past eruptions^9,11,12^, laboratory to large-scale experiments^13–15^, numerical modelling^16,17^, and a combination of these methods^18^. If adequate sedimentological models existed, the interpretation of preserved sediment would give all of the required interpretative information.

The hazard potential of PDCs, especially of the dilute type, which is the focus of this paper, depends in part on parameters such as dynamic pressure, (*P*_*dyn*_*)*, volumetric concentration of ash particles (*C)* and sedimentation rate (*S*_*r*_*)*_*,*_ here called impact parameters. Dynamic pressure^19^

1 $${P}_{dyn}=\frac{1}{2}{\rho }_{mix}{V}^{2},$$

where *V* is flow velocity and

2 $${\rho }_{mix}={\rho }_{s}C+{\rho }_{f}(1-C)$$

is gas-particle mixture density, contrasts the resistance of buildings to the flow^13^. Values lower than 1 kPa are not expected to cause damage to buildings^20^. The current starts breaking openings between 1 and 5 kPa, and at values higher than 5 kPa breaking of walls starts to occur. Particle volumetric concentration is a factor of hazard because ash particles in suspension in air are very harmful to breath^21,22^, even at temperatures lower than 200 °C and at concentration as low as 0.001 (that is typical of dilute PDCs), if exposure time is longer than a couple of minutes^21,23^. The sedimentation rate helps in defining hazard because it can be used for approximating flow duration (*t*) of dilute pyroclastic density currents^23,24^ (see the method section), which lasts until sedimentation is fed from turbulent suspension.

In this paper we use data from bedforms of dilute pyroclastic density currents for reconstructing the impact parameters and contribute to hazard assessment.

**Figure 1:**
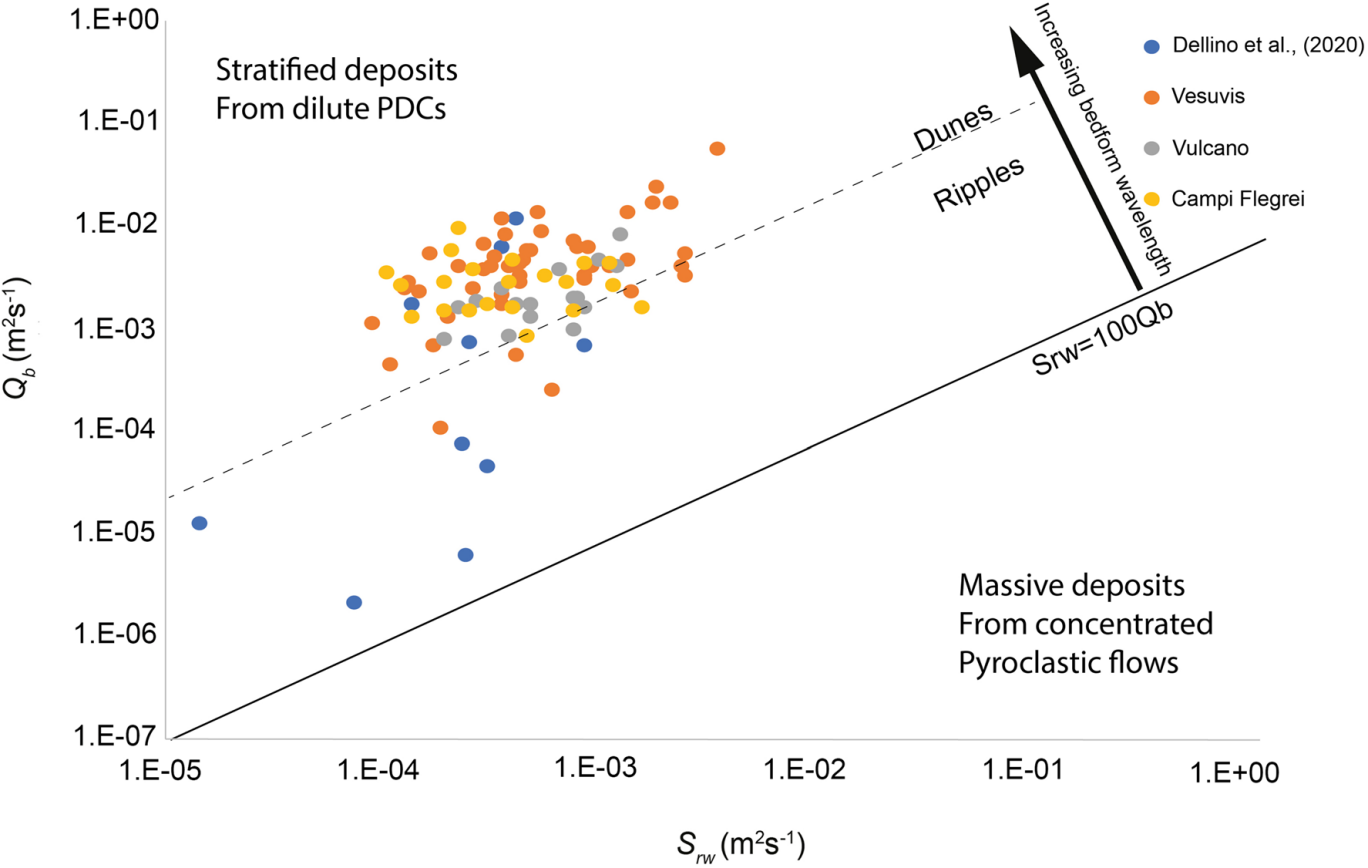
S_rw_ vs Q_b_ diagram in which 88 points have been added to those of Dellino et al.^30^, which are also included. The legend of volcanoes from which deposits were analysed is inserted. Modified after Dellino et al.^30^.

Bedforms of the types of dunes and ripples have been widely recognized in the deposits of dilute PDCs since the pioneering observations of Richards^25^, Moore^26^ and Fisher and Waters^27^. Further details on other types of bedforms are nowadays emerging from observation of recent eruptions^28,29^. Differently from what has been done for fluvial and turbiditic currents, only very few attempts have been made to construct phase diagrams defining the stability fields of bedforms as a function of PDCs flow regimes. Only very recently Smith et al.^12^ have proposed a phase diagram for highly concentrated volcanic granular currents. Douillet et al.^8^ carried wind tunnel experiments to relate the grain size of different types of volcanic particles to their threshold for motion at various slopes. Dellino et al.^30^ proposed a phase diagram (Fig. 1) in which volcanic deposits are classified according to the sedimentation rate and bedload transportation rate (*Q*_*b*_), which are modelled after large-scale experiments^13,31^. This agrees with the approach used in the field of sedimentary currents, where it is widely recognized that the proportion of bedload to suspended load and the sediment size are the major controlling factors on bedforms formation^5^. The experimentally validated model equations of the sedimentation and bedload transportation rates, on which the phase diagram of Fig. 1 is constructed, are defined and discussed in the method section. The lower right portion of the diagram represents the field of massive deposits, which are not considered further in this study. The upper portion of the diagram represents the field of stratified deposits with ripple and dune bedforms, which are related to highly expanded, fast-moving, dilute and turbulent PDCs, which are the focus of this paper. In the original version of the diagram, a link between the wavelength of bedforms and the ratio between the sedimentation and bedload transportation rates emerged, with ripples having a ratio larger than 0.05 and dunes smaller than 0.05. However, a well-defined correlation could not be found because of the paucity of data.

In this paper, we further populate the diagram in the portion of dilute PDCs by adding 88 points relative to various eruptions of Vesuvius, Campi Flegrei and Vulcano in Italy. With this addition, the new dataset consists of 98 deposits (Fig. 1) and covers a wide span of the sedimentation vs bedload transportation rates space, allowing a more detailed analysis of bedforms characteristics in terms of the flow parameters.

**Figure 2:**
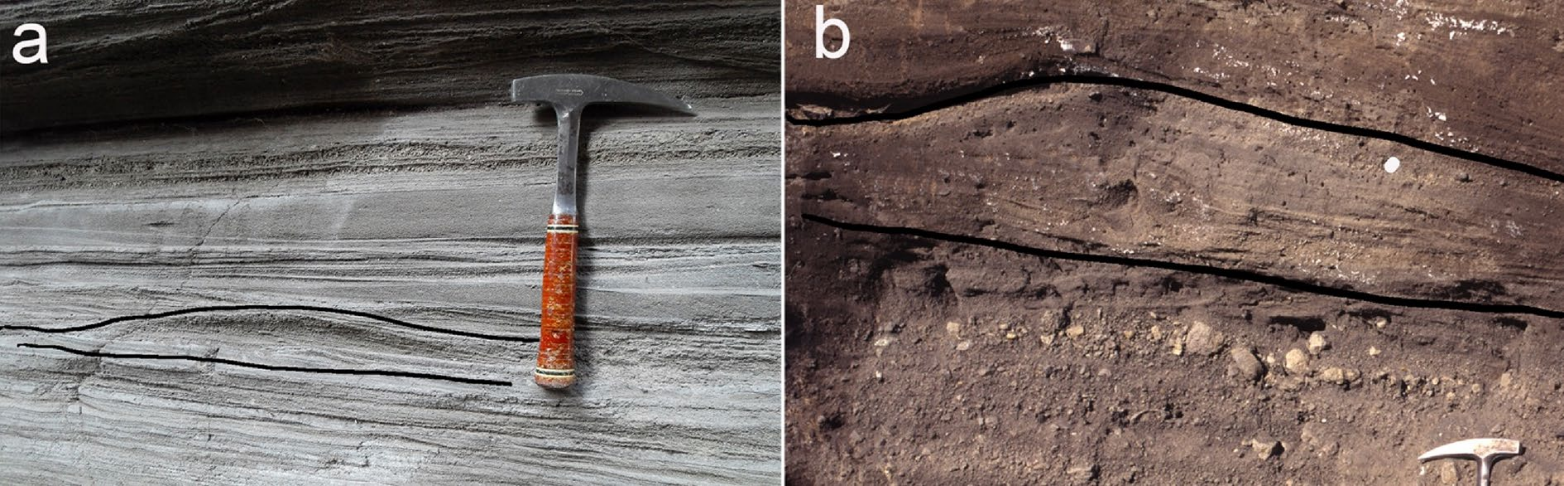
PDC deposits showing bedforms. (**a**) ripples of PDC deposits at Vulcano. The curves enclose a ripple with λ = 40 cm. (**b**) a dune bedform of PDC deposits at Vesuvius. The curves enclose a dune with λ = 200 cm.

In the enlarged dataset the bedform wavelength ranges from ripples (Fig. 2a), starting at 10 cm, to dunes (Fig. 2b), up to 250 cm.

We never found antidunes, in fact their interpretation has always been questioned in volcanic deposits^7,29,32^.

The software PYFLOW 2.0 by Dioguardi and Mele^33^ has been used to plot data in Fig. 1. It was implemented here so to obtain both the impact parameters of the current and also the sedimentation and bedload transportation rates, as defined by (11), (12) and (13) of the method section. The software employs sediment data that result from time-consuming laboratory analyses, which involve technologies and calculation resources not available to all scientists. The aim of this paper is to rearrange and simplify the dataset in order to construct a phase diagram where by means of deposits wavelength and grain size the impact parameters of dilute PDCs can be reconstructed directly in the field.

**Figure 3:**
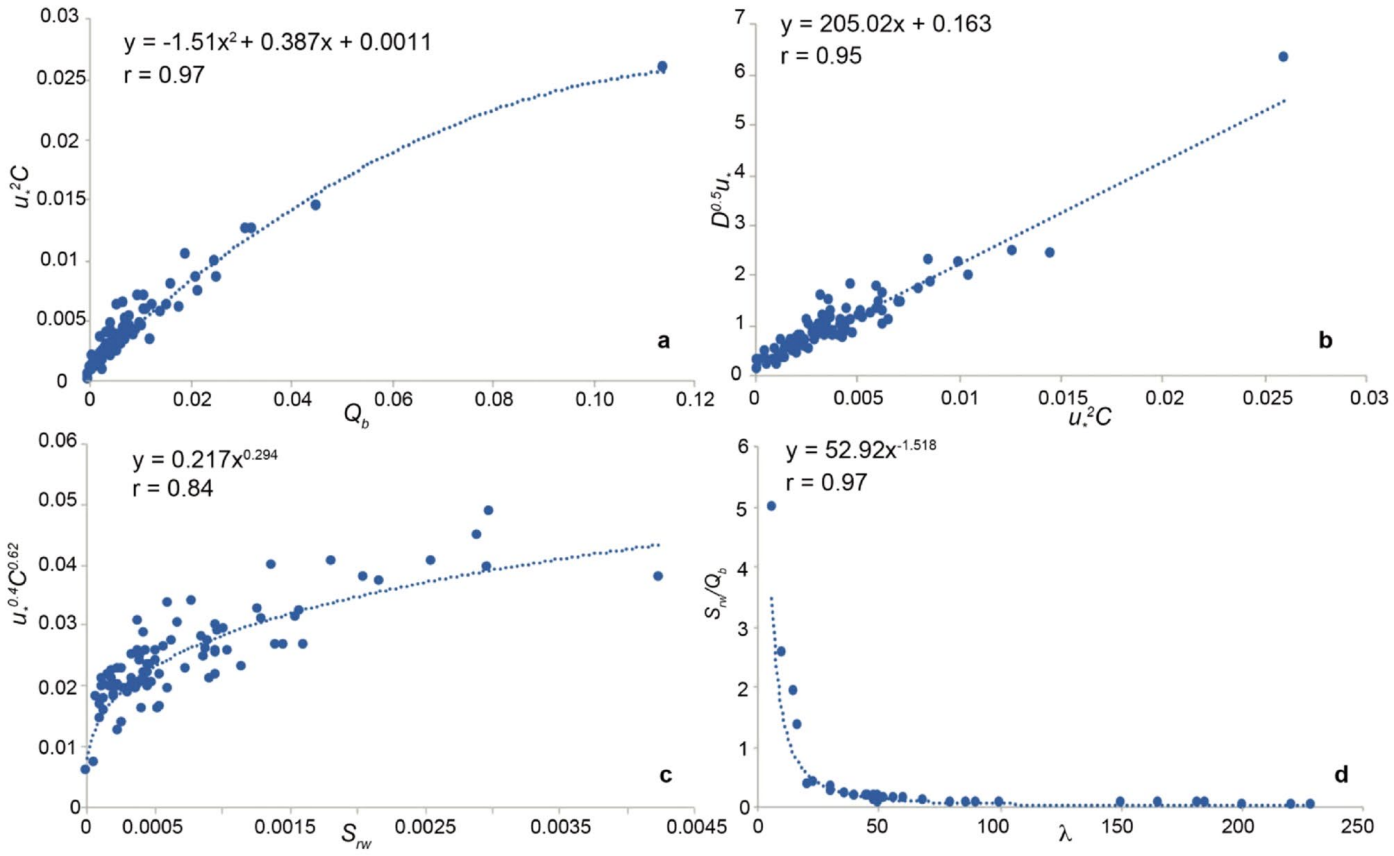
Fits resulting from the regression analysis. In the insets both the correlation coefficient, r, and the fitting equation are inset. (**a**) Parabolic relationship between ${\mathrm{u}}_{*}$^2^C and Q_b_. (**b**) linear relationship between D^0.5^${\mathrm{u}}_{*}$ and ${\mathrm{u}}_{*}$^2^C. (**c**) power-law relationship between ${\mathrm{u}}_{*}$^0.4^C^0.62^ and S_rw_. (**d**) power-law relationship between S_rw_/Q_b_ and λ.

Four fitting laws (Fig. 3), showing good correlation coefficients, were obtained by means of a regression analysis of the variables appearing in the equations of the sedimentation and bedload transportation rates. They represent relationships between four main fluid dynamic parameters of the current (namely shear velocity, ${u}_{*}$, particle volumetric concentration, sedimentation rate and bedload transportation rate) with bedform median grain size and wavelength. In the fitting laws many extra terms of the original equations do not appear anymore, thus allowing a reduction of the complexity of the formulas, and still evidencing the main characteristics of deposit formation process in terms of the current fluid dynamics.

A relationship between the bedload transportation rate and the product ${u}_{*}$ ^2^*C* is evidenced (Fig. 3a). Given that *C* is directly proportional to the density of the gas-particle mixture *(ρ*_*mix*_) and *ρ*_*mix*_ ${u}_{*}$^2^ is the turbulent shear stress of the current^34^ (*τ*), it implies that the bedload transportation rate is proportional to the shear stress, which confirms findings of sedimentary currents^29^. A relationship between *D*^*0.*5^${u}_{*}$ and ${u}_{*}$^2^*C* (Fig. 3b) implies that shear stress is proportional to bedforms median grain size, again in agreement with sedimentary deposits^30^. A relationship can be evidenced between the product of ${u}_{*}$^*0.*4^* C*^*0.624*^ and the sedimentation rate (Fig. 3c). Since the exponents of *C* and ${u}_{*}$ are both lower than 1, while in the fitting with the bedload transportation rate they are 1 and 2, respectively (Fig. 3a), it means that with an increase of *C* and ${u}_{*}$ the difference between the sedimentation rate and bedload transportation rate increases, and their ratio decreases. This justifies the decrease of the ratio as the bedform wavelength increases, as shown by the fitting of Fig. 3d. It gives a quantitative definition of the relationship between wavelength and the ratio between sedimentation and bedload transportation rates, which could not be precisely defined in the original diagram of Dellino et al.^30^. This new fitting has been obtained by plotting the wavelength, as measured in the field, of 32 deposits characterized by well exposed bedforms, against the value of the ratio of sedimentation and bedload transportation rates of the same deposits, as calculated by PYFLOW2. The continuity in the trend of Fig. 3d is at odd since ripples and dunes are not supposed to represent a continuum, being them separate by a hydrodynamic discontinuity^34^. Indeed, small ripples do not interfere with the upper current surface, while dunes, being larger, interfere with it. This discontinuity does not appear in the diagram of Fig. 3d, likely because a true interface between the current and the surrounding atmosphere does not exist in PDCs, at least in well-developed dilute currents, which are instead characterized by a very gradual passage between the two^6^ (see the method section for our model of dilute PDCs).

By means of the fitting laws, the following system of equations is formed:

$${u}_{*}^{2}C=1.5099{Q}_{b}^{2}+0.3874{Q}_{b}+0.0011$$

$${D}^{0.5}{u}_{*}=205.02{u}_{*}^{2}C+0.163$$

3 $${u}_{*}^{0.4}{C}^{0.62}=0.2168{S}_{r}^{0.2938}$$

$${S}_{rw}/{Q}_{b}=52.92{{W}_{l}}^{-1.518}$$

It can be solved numerically, once grain size (*D)* and wavelength (*λ*) are obtained in the field, and the current shear velocity ($${u}_{*})$$*,* particle concentration (*C*)*,* sedimentation rate *(S*_*rw*_) and bedload transportation rate (*Q*_*b*_), are obtained. The dynamic pressure, which is also relevant for the hazard of dilute PDCs, is subsequently calculated according to (1) and (2) by means of the particle volumetric concentration and shear velocity. The former is used for the calculation of flow velocity (*V*) as defined by the law of the wall of a turbulent boundary layer^34^

4 $$V\left(y\right)={u}_{*}\left(\frac{1}{k}ln\frac{y}{{k}_{s}}+8.5\right)$$

which is the physical model of PDCs that we employ (see the method section), where *V(y)* is the velocity profile of the stratified flow^34^, and *k*_*s*_ is the substrate roughness.

When comparing results obtained by (3) with those resulting from PYFLOW 2, the average absolute error of shear velocity is 28% and that of particle volumetric concentration is 30%, meaning that a good approximation of the two parameters can be achieved by means of the simplified formulas. In the case of sedimentation rate, the error is larger, about 45%, but still it is important to discuss its role as it allows to approximate the calculation of flow duration, *t*^23^, which is a main factor of hazard (see the method section).

**Figure 4:**
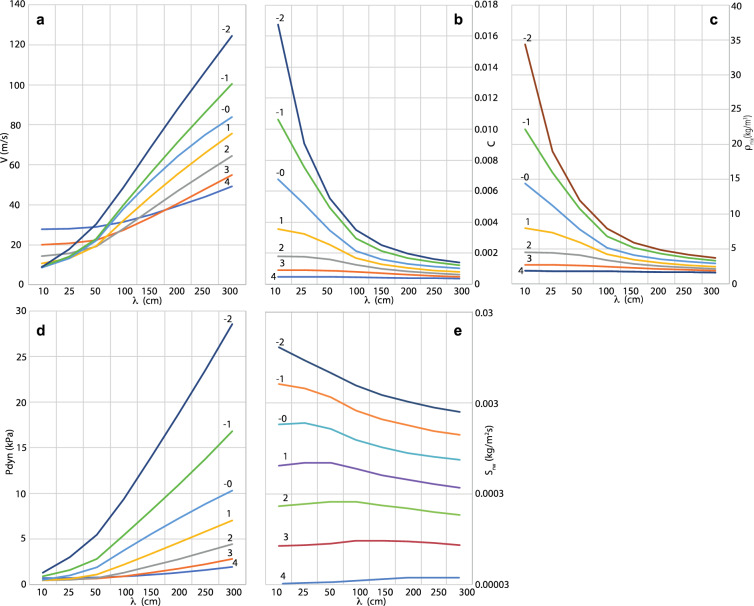
Phase diagrams showing the trends of flow variables and impact parameters of dilute PDCs as a function of bedform wavelengths and grain size. The various curves represent different grain size D. Grain size is expressed in phi units (phi = − log_2_D, with D in mm). (**a**) trend of velocity, V, height-averaged over the lowermost 1000 cm of the current in relation to λ. (**b**) trend of concentration C, in relation to λ. (**c**) trend of gas-particle mixture density ρ_mix_, in relation to λ. (**d**) trend of dynamic pressure, P_dyn_ in relation to λ. (**e**) trend of sedimentation rate S_rw_, in relation to λ.

As to show the relationship of bedforms’ median size and wavelength with current fluid dynamics and impact parameters, the system (3) was used to construct the phase diagrams of Fig. 4, where focus is put on bedforms of wavelength between 10 and 300 cm, although bedforms with larger wavelength can be found in the geologic record of volcanic deposits. The latter scenario is out of the range of applicability of our model and only bedforms that develop on an almost flat surface are considered here. Much larger bedforms typically develop as an interplay between the current’s flow dynamics and the ground morphology elements^29^. The range of median size (*D)* of Fig. 4 was set to typical grain-size values for laminated pyroclastic bedforms (between 4 and − 2 phi i.e. 0.0064 mm and 4 mm respectively) since larger sizes (coarse lapilli and bombs), which typically form lenticular beds, are thought to represent highly concentrated traction-carpets at the base of dilute PDCs^29,35,36^, to which our model does not apply.

The current velocity, *V,* of Fig. 4a, represents the average value of the lowest 1000 cm of the current and was obtained by integrating (4) over flow height, with k_s_ = 10 cm. We chose this depth-averaging height of the current because, in dilute PDCs, the portion responsible for the dynamic impact is the lowermost one (the shear flow) and 1000 cm represent a reasonable estimate of an average building height^37^. *V* increases at increasing *λ* but with different trends and rates depending on particle size *D*. In the diagram, velocity ranges from about 10 m/s to about 130 m/s. The trends are significantly different for the finer grain sizes (1 to 4 phi) at the smaller wavelengths (up to 50 cm), which can be interpreted as the smaller the wavelength, the smaller are the sedimentation and bedload transportation rates and shear stress, therefore the higher the velocity required to develop bedforms with the finer grain sizes. This is because for fine ash, due to the very low Reynolds number of shear (*R*_e*_), the initiation of motion at the bedload occurs at a very high critical Shield’s number ${(\theta }_{t})$^24,38^. Naturally, larger clasts have a higher threshold for motion, and thus involve a steeper shear stress gradient in the basal flow. This configuration increases the turbulence level, and thus the sediment discharge, allowing bedforms of longer wavelength to develop. The volumetric concentration, (*C)*, ranges from less than 0.001 to about 0.017 (Fig. 4b). It decreases as wavelength increases, and it does so for all grain sizes, although at a rate that decreases at decreasing grain size *D*, because a higher concentration favors a higher sedimentation rate (see (11) of the method section) and a larger ratio between sedimentation and bedload transportation rates, hence a smaller wavelength. The change in trend with decreasing grain size can be explained by the fact that the finer are is particles, the lower the concentration required to develop bedforms with small wavelengths. Current density, which was calculated by means of (2) and fixing *ρ*_*s*_ = 2000 kg/m^3^ and *ρ*_*f*_ = 0.9 kg/m^3^ (which is reasonable if the fluid, made up of volcanic gas plus entrained cold atmosphere, is at about 200 °C^23^) (Fig. 4c) follows the trend of concentration, and varies from less than 2 kg/m^3^ to about 35 kg/m^3^. The trend of dynamic pressure (*P*_*dyn*_*)* (Fig. 4d) varies from less than 1 kPa with smaller wavelengths and finer grain sizes, which is a value that does not cause severe damages to buildings^10,20^, to almost 30 kPa with larger wavelengths and coarser grain sizes, a value that can destroy even the more resistant, modern buildings of reinforced concrete^10,20^. The sedimentation rate (Fig. 4e) increases as grain size coarsens, meaning that with finer sizes flow duration is longer because smaller particles result in a smaller settling velocity. As far as the wavelength is concerned, for the finest sizes, the sedimentation rate increases at increasing wavelength, meaning a decrease of flow duration with longer bedforms. With the coarsest sizes, instead, the sedimentation rate decreases as wavelength increases, meaning a longer flow duration with longer bedforms.

The ranges of wavelength and grain size of Fig. 4 replicate the ranges of our dataset of dilute PDCs deposits, and result in impact parameters that span from currents that do not impact severely on structures, to values of devastating effects. Such a range well represents the situation of large-scale dilute PDCs whose strength decreases along runout^23^, and change from totally destructive flows around the volcano to residual currents that in the distal outreach do not possess a high strength but can still be rich in ash, as is the case of the dilute PDCs of the AD79 Vesuvius eruption at Pompeii^23^. Such fine material can be dangerous to breath even at concentrations lower than 0.001^39^, if flow duration lasts more than a couple of minutes.

As an operative help, to complement the diagrams of Fig. 4, the system (3) was solved at discrete intervals of grain size and wavelength, to construct a table where the stability fields of dynamic pressure, particle concentration and sedimentation rate, are represented inside a grid (Table 2). The values are averaged among the four neighboring grid points and the uncertainty is expressed in terms of one standard deviation. Dynamic pressure (*P*_*dyn*_) is calculated by considering the average value obtained by integration over the first 1000 cm of the current, and setting *k*_*s*_ = 10 cm and *ρ*_*s*_ = 2000 kg/m^3^.

**Table 2:** Phase diagram in which the stability fields of the impact parameters P_dyn_, C and S_rw_, are expressed as a function of λ and D of bedforms. The values inside the grid represent the average between the four neighboring grid points and the uncertainty is expressed as the standard deviation. ks = 10 cm, ρ_s_ = 2000 kg/m^3^.

*Wavelength (m)*	*Grain size (ϕ)*						
3	4	3	2	1	0	− 1	− 2
2.5	*Pdyn (kPa)*	2.14 ± 0.51	3.27 ± 0.96	5.21 ± 1.50	7.98 ± 1.98	12.4 ± 3.57	20.6 ± 6.66
	*C*	4.3E−4 ± 9.0E−5	5.9E−4 ± 9.7E−5	7.5E−4 ± 1.1E−4	9.5E−4 ± 1.5E−4	1.2E−3 ± 1.7E−4	1.4E−3 ± 1.7E−4
	*Srw (m*^2^*s*^−1^*)*	6.0E−5 ± 2.8E−5	1.3E−4 ± 5.7E−5	2.7E−4 ± 1.0E−4	5.5E−4 ± 2.1E−4	1.1E−3 ± 3.9E−4	2.0E−3 ± 6.6E−4
2	*Pdyn (kPa)*	1.72 ± 0.39	2.60 ± 0.79	4.18 ± 1.28	6.60 ± 1.84	10.2 ± 2.82	16.7 ± 5.55
	*C*	4.8E−4 ± 1.1E−4	6.7E−4 ± 1.2E−4	8.6E−4 ± 1.4E−4	1.1E−3 ± 1.8E−4	1.4E−3 ± 2.4E−4	1.7E−3 ± 2.3E−4
	*Srw (m*^2^*s*^−1^*)*	6.2E−5 ± 3.0E−5	1.4E−4 ± 6.3E−5	2.9E−4 ± 1.2E−4	6.0E−4 ± 2.3E−4	1.2E−3 ± 4.5E−4	2.2E−3 ± 7.7E−4
1.5	*Pdyn (kPa)*	1.35 ± 0.28	1.96 ± 0.63	3.19 ± 1.06	5.17 ± 1.63	7.93 ± 2.24	12.9 ± 4.51
	*C*	5.3E−4 ± 1.4E−4	7.8E−4 ± 1.6E−4	1.0E−3 ± 2.9E−4	1.3E−3 ± 2.3E−4	1.7E−3 ± 3.4E−4	2.1E−3 ± 3.4E−4
	*Srw (m*^2^*s*^−1^*)*	6.2E−5 ± 3.2E−5	1.5E−4 ± 7.2E−5	3.3E−4 ± 1.4E−4	6.8E−4 ± 2.7E−4	1.4E−3 ± 5.4E−4	2.6E−3 ± 9.2E−4
1	*Pdyn (kPa)*	1.04 ± 0.18	1.39 ± 0.46	2.23 ± 0.84	3.70 ± 1.37	5.68 ± 1.80	9.20 ± 3.55
	*C*	5.9E−4 ± 1.8E−4	9.3E−4 ± 2.4E−4	1.3E−3 ± 2.9E−4	1.7E−3 ± 3.6E−4	2.2E−3 ± 5.6E−4	2.8E−3 ± 5.9E−4
	*Srw (m*^2^*s*^−1^*)*	6.2E−5 ± 3.3E−5	1.6E−4 ± 8.3E−5	3.8E−4 ± 1.7E−4	7.9E−4 ± 3.2E−4	1.6E−3 ± 7.0E−4	3.2E−3 ± 1.2E−3
0.5	*Pdyn (kPa)*	0.81 ± 0.12	0.91 ± 0.30	1.34 ± 0.62	2.23 ± 1.09	3.47 ± 1.50	5.77 ± 2.69
	*C*	6.4E−4 ± 2.2E−4	1.1E−3 ± 3.7E−4	1.8E−3 ± 5.5E−4	2.4E−3 ± 7.5E−4	3.4E−3 ± 1.2E−3	4.2E−3 ± 1.2E−3
	*Srw (m*^2^*s*^−1^*)*	6.0E−5 ± 3.2E−5	1.7E−4 ± 9.0E−5	4.3E−4 ± 2.1E−4	9.8E−4 ± 4.5E−4	2.1E−3 ± 1.0E−3	4.2E−3 ± 1.7E−3
0.25	*Pdyn (kPa)*	0.68 ± 0.08	0.63 ± 0.09	0.76 ± 0.25	1.16 ± 0.53	1.82 ± 0.77	3.22 ± 1.63
	*C*	6.7E−4 ± 2.5E−4	1.3E−3 ± 4.6E−4	2.3E−3 ± 7.5E−4	3.6E−3 ± 1.1E−3	5.3E−3 ± 1.7E−3	6.8E−3 ± 1.9E−3
	*Srw (m*^2^*s*^−1^*)*	5.7E−5 ± 3.0E−5	1.6E−4 ± 8.9E−5	4.5E−4 ± 2.4E−4	1.2E−3 ± 5.8E−4	2.8E−4 ± 1.3E−3	5.7E−3 ± 2.4E−3
0.1	*Pdyn (kPa)*	0.64 ± 0.09	0.53 ± 0.04	0.53 ± 0.08	0.65 ± 0.24	0.99 ± 0.43	1.70 ± 0.92
	*C*	6.9E−4 ± 2.5E−4	1.3E−4 ± 5.0E−4	2.6E−4 ± 9.4E−4	4.7E−3 ± 1.6E−3	7.5E−3 ± 2.3E−3	1.1E−2 ± 4.0E−3
	*Srw (m*^2^*s*^−*1*^*)*	5.6E−5 ± 2.9E−5	1.5E−5 ± 2.8E−5	4.3E−4 ± 2.4E−4	1.2E−3 ± 6.5E−4	3.1E−3 ± 1.6E−3	7.5E−3 ± 3.7E−3
	0.0064	0.0125	0.250	0.5	1	2	4

In the supplementary file, additional tables with *k*_*s*_ = 10 cm and *ρ*_*s*_ = 1000 kg/m^3^; *k*_*s*_ = 30 cm and *ρ*_*s*_ = 2000 kg/m^3^; and *k*_*s*_ = 10 cm and *ρ*_*s*_ = 1000 kg/m^3^ are included (Supp. Tables 1, 2 and 3 respectively), and an additional table is provided (Supp. Tab. 4) where the values of ${u}_{*}$, *C* and *S*_*r*_ are set at half phi intervals of *D* in relation to *λ*. By means of these data, and specifying in (1) and (4) the value of *ks*, *ρ*_*s*_, and *H* at which to integrate *V*, more precise data of the impact parameters can be obtained.

With our diagrams and tables at hand it is thus possible to invert bedforms of past eruptions, and reconstruct the impact parameters of dilute pyroclastic density current, contributing to hazard assessment. Various approximations are introduced in our model as, for example, a fixed average density of particles, which in fact can be highly variable among the components of the grain-size mixture, or the median value as representing a single characteristic value of the grain-size distribution, which in some cases can be quite complex. Due to these approximations, we recommend to carefully consider the uncertainty ranges of Table 2 (and of the supplementary tables) when judging the values of impact parameters. Furthermore, it is true that bedforms are not always well exposed in their complete longitudinal profile, because of truncations due to erosion. Sometimes they are also difficult to measure precisely, because a direct access to the deposit is hard. However, in the case that these can be accessed, well-preserved bedforms are a common signature of dilute PDC deposition. In such case our model can be used for quantifying the impact parameters of dilute pyroclastic density currents and contribute to the hazard assessment of active volcanoes.

## Method

**Figure 5:**
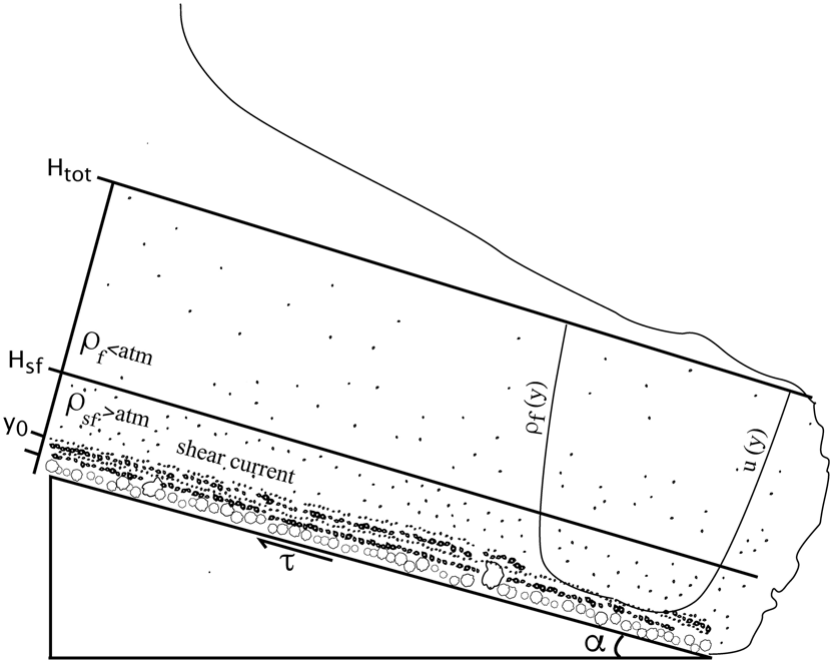
Sketch of the model of a well-established dilute pyroclastic density current used in this paper (modified after Dellino et al.^37^).

The reconstruction of the impact parameters of PDCs is based on a flow dynamics model that starts with the assumption that the turbulent current is velocity and density stratified^19,37^. In the stratified multiphase gas-particle current, the basal part is a shear flow that moves attached to the ground and has a density higher than atmosphere (Fig. 5). The upper part is buoyant, because particle concentration decreases with height down to a value that, combined with the effect of gas temperature, makes the mixture density lower than the surrounding atmosphere.

In a PDC, particles are mainly transported by turbulent suspension and sedimentation is controlled by a balance between flow shear velocity u_*_, which is controlled by fluid turbulence and favors suspension, and particle settling velocity, *w*_*t*_ = *(4gD(ρ*_*s*_*—ρ*_*mix*_*)/3C*_*d*_*ρ*_*mix*_*)*^*0.*5^, which favors sedimentation, where *C*_*d*_ is drag coefficient. During sedimentation, it is assumed that particles of different composition, i.e. crystals and glass, settle at the same aerodynamic conditions, e.g., with the same terminal fall velocity^37^. Therefore, by equating the settling velocity of the glass and crystal components in the deposit, and assuming that sedimentation starts when *P*_*n*_ = 2.5^40^, hence when *w*_*t*_ = u_*_, flow shear velocity and density *ρ*_*sf*_ of the shear flow can be calculated after *D*, *ρ*_*s*_ and *C*_*d*_ are measured in the laboratory^37,41^. These are the main input data in the PYFLOW_2.0 code^33^, which allows reconstructing the current parameters.

The code is based on a model that assumes PDCs behave as turbulent boundary layer shear flows moving over a rough surface^37^, which velocity profile is given by (9). The model has been validated by experiments^13^ and already applied to other eruptions^42^. Here it is summarized as adapted from Dellino et al.^23^.

The maximum volumetric concentration of particles that can be transported in turbulent suspension, i.e. the maximum current capacity, is a function of the Rouse number of the particulate mixture taken in suspension. The profile of volumetric concentration over current height is regulated by the Rouse model^40^.

5 $$C\left(y\right)={C}_{0}{(\frac{{y}_{0}}{H-{y}_{0}}\frac{H-y}{y})}^{{P}_{n}}$$

where *C*_*0*_ is the particle volumetric concentration at a reference height *y*_*0*_ and *H* is the total current thickness. Assuming steady sedimentation, *H* is obtained by the ratio *H*_*dep*_*/C*_*sf*_ where *H*_*dep*_ is deposit thickness and *C*_*sf*_ is the depth-averaged concentration in the basal shear flow, which can be calculated by *ρ*_*sf*_ = *ρ*_*s*_* C*_*sf*_ + *ρ*_*f*_*(1-C*_*sf*_*),* when *ρ*_*sf*_ and *ρ*_*f*_ are known.

The shear-flow height and density are obtained by solving the system of (6) and (*7*), which is valid for a turbulent current

6 $$\tau =\left({\rho }_{sf}-{\rho }_{f}\right)gsin\alpha {H}_{sf}$$

7 $$\tau ={\rho }_{sf}{u}_{*}^{2}$$

where $\tau$ is the shear-driving stress of the flow moving down an inclined slope of angle $\alpha$.

The density profile, which is a function of concentration, particle density and gas density, is:

8 $${\rho }_{mix}\left(y\right)={\rho }_{f}+{C}_{0}{\left(\frac{{y}_{0}}{H-{y}_{0}}\frac{H-y}{y}\right)}^{{P}_{n}}\left({\rho }_{s}-{\rho }_{f}\right)$$

The gas density and Rouse number are obtained by solving numerically the following system:

9 $${\rho }_{a}\left(y\right)={\rho }_{f}+{C}_{0}{\left(\frac{{y}_{0}}{H-{y}_{0}}\frac{H-{H}_{sf}}{{H}_{sf}}\right)}^{{P}_{n}}\left({\rho }_{s}-{\rho }_{f}\right)$$

10 $${\rho }_{sf}=\frac{1}{{H}_{sf}-{y}_{0}}\underset{{y}_{0}}{\overset{{H}_{sf}}{\int }}\left({\rho }_{f}+{C}_{0}{\left(\frac{{y}_{0}}{H-{y}_{0}}\frac{H-y}{y}\right)}^{{P}_{n}}\left({\rho }_{s}-{\rho }_{f}\right)\right)dy$$

Equation (9) states that atmospheric density, ${\rho }_{a}$, is reached at the top of the shear flow, *H*_*sf*_, and Eq. (10) states that the average density of the shear flow, ${\rho }_{sf}$ refers to the part of the flow that goes from the reference level, *y*_*0*_, to the shear flow top height, *H*_*sf*_.

By combining the velocity and density profiles, the dynamic pressure profile is finally obtained. The profiles of the flow parameters are expressed in terms of a probability density function that depends on the variance of particle characteristics.

In dilute PDCs, sedimentation occurs by continuous aggradation during the passage of the current, and the total time of aggradation is a proxy of flow duration, *t*^23^, which is equal to deposit thickness, *H*_*dep*_*,* divided by the aggradation rate ${A}_{r}$. The latter is equal to *S*_*rw*_ divided by one meter, which is the reference width of the sedimentation rate per unit width, (see Dellino et al.^30^). Therefore, flow duration, which approximates the time in which harmful concentrations of ash are suspended in the current to which a human being can be exposed, can be calculated by means of *S*_*rw*_.

The software has been here implemented, therefore, as to obtain also the sedimentation rate (*S*_*r*_) as defined by the experimental model of Dellino et al.^24^, as:

11 $${S}_{r}=\left(\sum_{i}^{n}{\rho }_{{s}_{i}}{w}_{{t}_{i}}\left(\frac{\frac{{\phi }_{i}/{\rho }_{{s}_{i}}}{\sum_{i=1}^{n}{\phi }_{i}/{\rho }_{{s}_{i}}}*C}{\left(\left(10.065*\frac{{P}_{ni}}{{P}_{n}^{*}}\right)+0.1579\right)}*0.7+\frac{\frac{{\phi }_{i+1}/{\rho }_{{s}_{i}+1}}{\sum_{i=1}^{n}{\phi }_{i+1}/{\rho }_{{s}_{i+1}}}*C}{\left(\left(10.065*\frac{{P}_{ni}}{{P}_{n}^{*}}\right)+0.1579\right)}*0.3\right)\right)-0.01$$

with the subscript *i* referring to the *ith* particle-size class and *n* being the number of size classes of the grain-size distribution of the sediment, where *P*_*ni*_ = *w*_*ti*_/ku_*_ is the Rouse number of the *i*_*th*_ size fraction of the solid material suspended in the current, with *k* the Von Karman constant (0.4) and *w*_*ti*_ the terminal velocity of the *i*_*th*_ size fraction. *P*_n_^*^ = *P*_*navg*_*/P*_*nsusp*_ is the normalized Rouse number of the current, i.e. the ratio between the average Rouse number of the solid material in the current and the Rouse number at maximum suspension capacity. ${\phi }_{i}$, ${\rho }_{{s}_{i}}$ and ${P}_{ni}$ are the weight fraction, the density and the Rouse number of the *i*_*th*_ grain-size fraction, respectively. The sedimentation rate was transformed in the sedimentation rate per unit width, *S*_*rw*_ in order to make it comparable with *Q*_*b*_ dimension^30^.

The software has been also implemented with the calculation of the bedload transportation rate (*Q*_*b*_) by the use of the experimental formulation of Dellino et al.^30^:

12 $${Q}_{b}=\sum_{i}^{n}{q}_{bi}$$

where

13 $${q}_{bi}=\frac{({\rho }_{s}/{\rho }_{mix}-1)g{q}_{bi}}{{W}_{i}^{*}{\phi }_{i}{u}_{*}^{3}}\mathrm{ and }\,{W}_{i}^{*}=\left\{\begin{array}{c}0.002{\xi }^{7.5}\,for\,\xi <1.35\\ 14\left(1-\frac{0.894}{{\xi }^{0.5}}\right)\,for\,\xi \ge 1.35\end{array}\right.$$

${q}_{bi}$ is the volumetric bedload transport rate of the *i*_*th*_ size fraction per unit width of the flow, and $\xi =\tau /{\tau }_{ri}$ is the normalized shear stress, where ${\tau }_{ri}$ is the minimum shear stress needed to move the *i*_*th*_ size fraction at bedload.

## Supplementary Information

Supplementary Information.
