# Supplementary material for: Inverting sediment bedforms for evaluating the hazard of dilute pyroclastic density currents in the field
Source: Sci Rep. 2021 Oct 25;11:21024. doi: 10.1038/s41598-021-00395-3 (PMC8545956; doi:10.1038/s41598-021-00395-3)
Supplement: Supplementary file 1 — Supplementary Information. [file 41598_2021_395_MOESM1_ESM.docx]

*Supplementary Table 1. Phase diagram in which the stability fields of the impact parameters P_dyn_, C and S_rw_, are expressed as a function of λ and D of bedforms. The values inside the grid represent the average between the four neighboring grid points and the uncertainty is expressed as the standard deviation. ks = 10 cm, ρ_s_ = 1000 kg/m^3^.*

**

*Supplementary Table 2. Phase diagram in which the stability fields of the impact parameters P_dyn_, C and S_rw_, are expressed as a function of λ and D of bedforms. The values inside the grid represent the average between the four neighboring grid points and the uncertainty is expressed as the standard deviation. ks = 30 cm, ρ_s_ = 1000 kg/m^3^.*

**

*Supplementary Table 3. Phase diagram in which the stability fields of the impact parameters P_dyn_, C and S_rw_, are expressed as a function of λ and D of bedforms. The values inside the grid represent the average between the four neighboring grid points and the uncertainty is expressed as the standard deviation. ks = 30 cm, ρ_s_ = 2000 kg/m^3^.*
